# PLGA Microspheres Incorporated Gelatin Scaffold: Microspheres Modulate Scaffold Properties

**DOI:** 10.1155/2009/143659

**Published:** 2009-03-30

**Authors:** Indranil Banerjee, Debasish Mishra, Tapas K. Maiti

**Affiliations:** Department of Biotechnology, Indian Institute of Technology, Kharagpur, West Bengal 721302, India

## Abstract

Freeze drying is one of the popular methods of fabrication for poly(lactide-co-glycolide) (PLGA) microspheres incorporated polymer scaffolds. However, the consequence of microspheres incorporation on physical and biological properties of scaffold has not been studied yet. In this study, attempt has been made to characterize the effect of PLGA microsphere incorporation on the physical properties of freeze-dried gelatin scaffold and its influence on cytocompatibility. Scaffolds loaded with varying amount of PLGA microspheres (10%, 1%, 0.1% w/w) were subjected to microarchitecture analysis, swelling, porosity, mechanical properties, biodegradation, cell adhesion, and cell proliferation studies. Results revealed that an increase in percentage loading of microspheres reduced the pore size and uniformity of the pore structure. Moreover, loading of PLGA microspheres up to 1% w/w significantly increased porosity, swelling, and mechanical properties of the scaffold but variations were not proportional for 10% w/w loading. Results also showed that PLGA microspheres have no significant effect on cell adhesion but influenced the growth kinetics.

## 1. Introduction

Freeze drying is one of the popular methods of scaffold formation in tissue engineering. This is a method of choice especially for the preparation of natural polymeric scaffold like gelatin, chitosan, collagen, and so forth [1–3]. Freeze-drying method involves the formation of ice crystals inside polymer solution during freezing, and those ice crystals act as porogens during lyophilization that results a porous three-dimensional polymer scaffold. In case of freeze drying, any physical or chemical factors that can change the pattern of ice crystal formation and distribution (e.g., freezing temperature [4]) can change scaffold microarchitecture.

Recently, effort has been made to fabricate a smart controlled release tissue engineering matrix by incorporating therapeutics loaded microspheres into polymer solution followed by freeze drying [5]. Objective of such efforts is the efficient and controlled delivery of therapeutic molecules during tissue remodeling and regeneration [6–10]. Among all these microsphere-based controlled release systems, PLGA microspheres have got an edge over the others because of its biocompatibility and known efficiency to deliver a number of growth factors, proteins, or drugs in a time dependent manner both in vitro and in vivo [11–13]. A couple of literatures have clearly stated that therapeutics molecule loaded PLGA microsphere embedded scaffold can be fabricated by freeze drying, and those scaffolds can act as a controlled release engineered matrix. In all these efforts, the main aim was to achieve the desired therapeutic benefit but variation in physical properties of the scaffold with PLGA microspheres incorporation was not properly characterized [14–16]. This overlooked phenomenon may be crucial for the stability and performance of PLGA microspheres loaded freeze-dried natural polymeric scaffold. PLGA is hydrophobic as compared to many natural biopolymers like gelatin, alginate [17–19]; therefore, presence of PLGA microspheres inside the hydrophilic polymers at the time of freezing can alter the size and distribution of ice crystals formed and such alteration can change the microarchitecture of the freeze-dried scaffold. Furthermore, microspheres may cause changes in the mechanical properties of the scaffold [20]. In this present work, an attempt has been taken to characterize the effect of PLGA microspheres incorporation on physical properties of freeze-dried scaffold and its impact on the performance of the cells cultured on the scaffold. For this purpose, PLGA (65:35) microspheres loaded gelatin scaffold made by freeze drying was chosen as model. Gelatin is widely used in tissue engineering as scaffold materials because of its biocompatibility, low immunogenicity, and biodegradability [21]. Influence of PLGA microsphere incorporation on the physical properties of the freeze-dried gelatin scaffold was studied by analyzing the changes in scaffold microarchitecture, porosity, swelling, mechanical strength and biodegradation with varying amount of PLGA microspheres loading. Impact of PLGA microsphere incorporation in the above mentioned scaffolds on cellular performance was further characterized by studying the adhesion and growth kinetics of murine fibroblast L929 cells on these scaffolds.

## 2. Materials and Methods

Gelatin, glutaraldehyde, poly(DL-lactide-co-glycolide) PLGA (65:35) were purchased from Sigma-Aldrich (St. Louis, MO, USA). DMEM was from GibcoBRL (Grand Island, NY, USA). FBS was obtained from Hyclone (South Logan, UT, USA). Murine L929 cells were obtained from NCCS Pune, India. Other chemicals used were purchased from local vendors.

### 2.1. Preparation of PLGA and Gelatin Film

A 5% (w/v) PLGA (65:35) solution in dichloromethane was prepared and cast into Petri dishes followed by solvent evaporation at room temperature. The film formed was vacuum dried for 48 hours and kept in desiccators until further use. In case of preparation of gelatin film, 3% gelatin solution was crosslinked with glutaraldehyde (0.05%) and cast on Petri dish for film formation.

### 2.2. Contact Angle Measurement of PLGA and Gelatin Film

To keep an account of hydrophobicity of the two polymers used in this model, the advancing contact angles of three replicates of PLGA (65:35) and gelatin (3% w/v, glutaraldehyde crosslinked) films were determined using a dynamic contact angle meter and tensiometer (model: D CAT, 11 DataPhysics). Briefly, each sample was attached to a microbalance and immersed into the wetting medium (deionised water). The wetting force at the solid/liquid/vapor interface was automatically recorded via an electrobalance as function of both time and immersion depth; this was converted into the advancing contact angle.

### 2.3. Preparation of PLGA Microspheres

PLGA microspheres were prepared using emulsion-solvent evaporation method. In brief, 200 mg of PLGA (65:35) was dissolved in 4 mL methylene chloride. The solution was mixed with 20 mL aqueous solution of 1% poly vinyl alcohol (PVA) and sonicated using an ultraprobe adopting regular pattern of ultrasonic vibration for 30 seconds followed by a pause of 30 seconds thrice (at 15 watt) in an ice bath. The resulting emulsion was stirred for 3 hours at room temperature followed by methylene chloride evaporation. The microsphere prepared in this way was collected by centrifugation at 10000 g. Then, it was washed thrice with PBS to remove excess PVA and finally lyophilized to get powder.

### 2.4. Characterization of Microsphere

Lyophilized powdered microspheres were examined by a scanning electron microscope (model: JEOL JSM-5800). Prior to observation, samples were sputter coated with gold, and the imaging was conducted at an accelerating voltage of 10 kV. At least 100 particles were examined to get average diameter and particle size distribution.

### 2.5. Preparation of PLGA Microspheres Incorporated Gelatin Scaffolds

Aqueous suspension of PLGA microspheres 0.1% (w/v) was mixed proportionally to 3% gelatin solution under constant stirring to prepare three different blends of PLGA and gelatin (10%, 1%, 0.1% PLGA microspheres with respect to gelatin weight). 2.5 mL of each suspension was cast in Petri dish (60 mm diameter) in presence of 0.05% glutaraldehyde. All the resulting suspensions were allowed to crosslink for 12 hours at room temperature. The crosslinked hydrogels were frozen at −20°C for 12 hours followed by 24 hours lyophilization.

### 2.6. Study of Scaffold Morphology

Scaffold morphology was analyzed using phase contrast microscope (Olympus CK X 41) and scanning electron microscope for elucidation of influence of PLGA incorporation on scaffold microarchitecture. Prior to observation through scanning electron microscope scaffolds were sputter coated with gold and analyzed at an accelerating voltage of 20 kV. The objective of the study was to characterize distribution of microspheres inside the scaffold and the effect of microsphere incorporation on pore size of the scaffold. For each analysis, at least 50 pores were examined.

### 2.7. Study of the Water Uptake Ability (Swelling Test)

Effect of microsphere incorporation on water absorption capacity was determined by swelling the scaffolds in water at room temperature. A known weight of scaffold material was placed in water and after 24 hours incubation, its wet weight was determined. The percentage water absorption (*W*sw) of the scaffold was calculated from the expression

(1) $$W\text{sw}=[\frac{\left(W_{24\text{ h}}-W_{0}\right)}{W_{0}}]\times 100,$$

where *W*_24 h_ represents the wet weight of scaffold after 24 hours of incubation, and *W*_0_ is the initial weight of the scaffolds. The values were expressed as mean ± SD (*n* = 3).

### 2.8. Porosity Analysis

Variation in porosity of the scaffold due to microspheres loading was determined using a mercury intrusion porosimeter (Poremaster, Quantachrome). In brief, scaffolds were degassed under vacuum and placed inside the penetrometer. Analysis was done at low pressure using mercury keeping 10-second equilibration time at each pressure step.

### 2.9. Study of Mechanical Properties

Mechanical properties of the scaffolds were tested using a universal testing machine (Hounsfield H25kS, Surrey, England). 30 × 10 × 1 mm scaffold pieces were subjected for tensile strength measurement at dry condition using a cross-head speed 1 mm/min. Tensile strength and percentage elongation at break were recorded. Data were analyzed using Q.MAT 3.1 software. The values were expressed as mean ± SD (*n* = 3).

### 2.10. Biodegradation Study

To study the effect of microspheres loading on in vitro biodegradation, scaffolds were incubated in PBS (pH 7.4) for 10 days at 37°C. The biodegradation was calculated in terms of percentage (%) weight loss using the formula

(2) $$[\frac{\left(W_{0}-W_{n}\right)}{W_{0}}\times 100],$$

where *W*_*n*_ is the dry weight of scaffold after “*n*” days incubation in PBS, and *W*_0_ is its initial weight. The values were expressed as the mean ± SD (*n* = 3).

### 2.11. Cell Adhesion Study

Cell adhesion study on the scaffolds was performed using mouse fibroblast L929 cells. In brief, murine L929 cells were cultured in DMEM containing 10% FBS in a 5% CO_2_ incubator at 37°C. At confluence, cells were harvested from the flask by trypsinization, and 5 × 10^4^ cells/cm^2^ were seeded on each scaffold of 1 cm × 1 cm × 0.1 cm dimension. Cells were allowed to adhere on the scaffold at 37°C for 4 hours. Cell adhesion on the scaffold was assessed by MTT method [22]. The values were expressed as mean ± SD (*n* = 3).

### 2.12. Cell Proliferation Study

Mouse fibroblast L929 cells were cultured in DMEM containing 10% FBS in a 5% CO_2_ incubator at 37°C. At confluence, cells were harvested from the flask by trypsinization, and 4 × 10^5^cells/cm^2^ were seeded on each type of scaffolds of 1 cm × 1 cm × 0.1 cm dimension. Scaffolds seeded with cells were kept days in 5% CO_2_ incubator at 37°C, and cells were allowed to grow up to 7 days. Media was replaced in each alternative day. After definite intervals, scaffolds were taken out and cell growth was estimated using MTT assay. All the experiments were done in triplicate.

### 2.13. Study of Cell Morphology

To check the influence of incorporated PLGA microspheres on cell growth and cell morphology, cell-seeded scaffold (after 3 days of initial seeding) was taken and subjected for analysis using scanning electron microscopy. Cells were fixed with 2.5% glutaraldehyde for 4 hours at room temperature, and it was then serially dehydrated using alcohol, sputter coated with gold, and then examined by a scanning electron microscope (model: JEOL JSM-5800).

### 2.14. Statistical Analysis

Experiments were run in triplicate for each sample. All data were expressed as mean ± standard deviation (SD) for *n* = 3. Student's *t*-test analysis was done to assess the statistical significance of the data sets.

## 3. Results

### 3.1. Contact Angle Measurement of PLGA and Gelatin Film

Contact angle is a measure of hydrophobicity of a material. The higher the contact angle is, the higher the hydrophobicity of the material is. Here, the advancing contact angle measured for gelatin film was 55.2 ± 1.3° and that of PLGA film was 71.2 ± 0.4°. This result confirms the significant difference in the hydrophobicity of two used polymers.

### 3.2. Characterization of PLGA Microsphere

**Figure 1:**
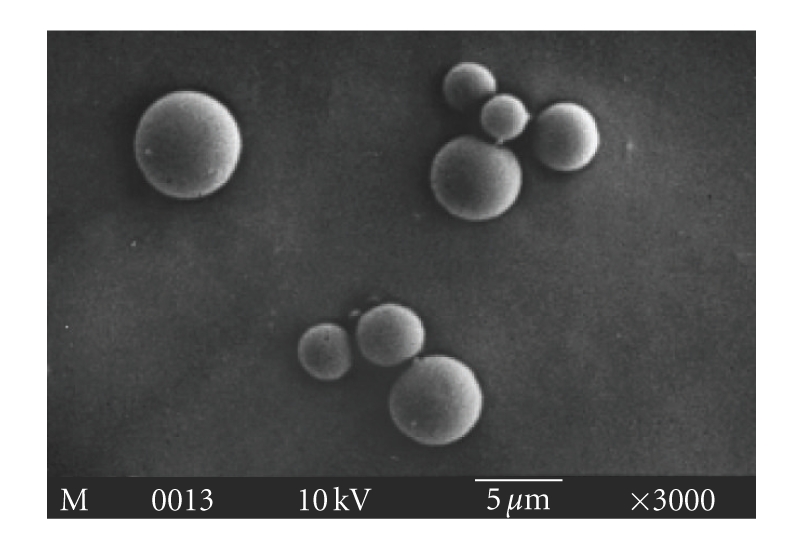
Scanning electron micrograph of PLGA microsphere formed by emulsion-solvent evaporation method.

The PLGA microspheres were prepared by emulsion—solvent evaporation method. The microspheres were spherical in shape and had a smooth surface as judged by SEM (see Figure 1). Size distribution of microsphere was found in the range of 1–15 *μ*m (96% of total population among which 53% were in the range of 2–5 *μ*m (data not shown)).

### 3.3. Microarchitecture of PLGA Microsphere Loaded Gelatin Scaffold

**Figure 2:**
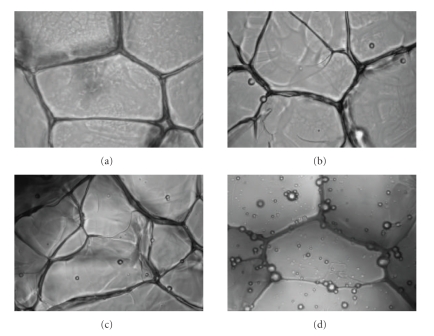
Phase contrast micrograph of PLGA microsphere incorporated gelatin scaffolds. (a) Pure gelatin scaffold, (b) 0.1% w/w PLGA microsphere incorporated scaffold, (c) 1% w/w PLGA microsphere incorporated scaffold, and (d) 10% w/w PLGA microsphere incorporated scaffold. Photographs were taken at 40X.

**Figure 3:**
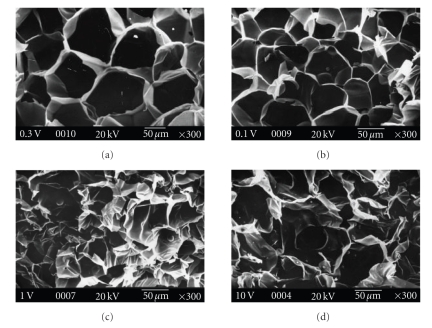
Scanning electron micrograph of microspheres incorporated gelatin scaffolds. (a) Pure gelatin scaffold, (b) 0.1% w/w PLGA microsphere incorporated scaffold, (c) 1% w/w PLGA microsphere incorporated scaffold, and (d) 10% w/w PLGA microsphere incorporated scaffold.

It is evident from the phase contrast and SEM micrographs (see Figures 2 and 3) that PLGA microspheres were uniformly distributed through out the gelatin matrix irrespective of the amount of microsphere added. Incorporation of PLGA microspheres during fabrication of gelatin scaffold by freeze—drying has significant effect on the overall microarchitecture of the scaffold. Effect of microsphere doping on scaffold pore size was summarized in Table 1. Among these four different scaffolds, control gelatin scaffold has regular pore structure (average pore diameter 160 *μ*m). Loading of 0.1% microspheres reduced the average pore size to 110 *μ*m. In case of 1% w/w PLGA microsphere loaded scaffold, there were two set of pores (average diameter 150–120 *μ*m which is 60% of total abundance and average diameter 50–30 *μ*m which is 40% of total abundance). 10% w/w PLGA microsphere loaded scaffold has no regular pore structure (pore diameter varied from 30–150 *μ*m).

### 3.4. Swelling Property

**Figure 4:**
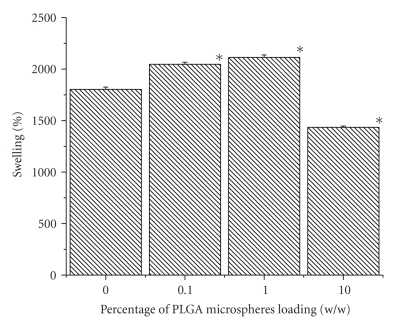
Effect of PLGA microsphere incorporation on the swelling property of gelatin scaffold. Values are mean ± S.D. (*n* = 3). *P < .005, compared to gelatin scaffold having no PLGA microsphere.

The water uptake ability of the scaffolds was in the range of 1400–2100% (see Figure 4). It was expected that incorporation of hydrophobic PLGA microsphere would reduce the water uptake in a dose dependent manner. However, no such trend was observed. Result showed that 1% w/w PLGA microspheres loaded scaffold has the highest swelling properties (2144%), and 10% PLGA microsphere loaded scaffold has shown the least swelling of (1435.33%).

### 3.5. Porosity

**Figure 5:**
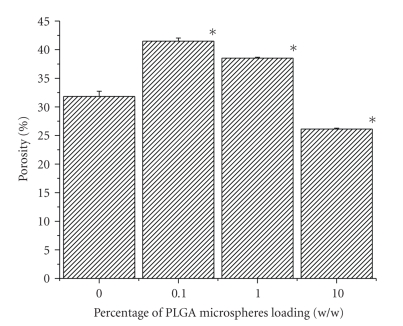
Effect of PLGA microsphere incorporation on scaffold porosity. Values are mean ± S.D. (*n* = 3). *P < .05, compared to gelatin scaffold having no PLGA microsphere.

All the scaffolds had porosity in the range of 25 to 42 percent (see Figure 5). Porosity of 10% PLGA microsphere loaded scaffold (26.13%) is significantly less than that of control (31.83%), where 0.1% and 1% w/w PLGA scaffold has porosity greater than control (41.5% and 38.53%, resp.).

### 3.6. Mechanical Properties of the Scaffold

**Figure 6:**
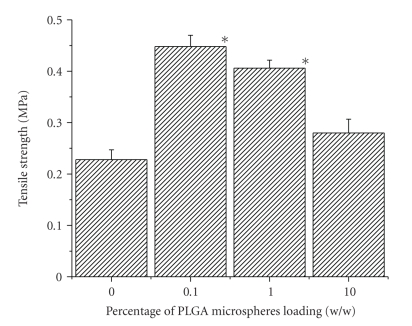
Effect of PLGA microsphere incorporation on the tensile strength of gelatin scaffold. Values are mean ± S.D. (*n* = 3). *P < .05, compared to gelatin scaffold having no PLGA microsphere.

**Figure 7:**
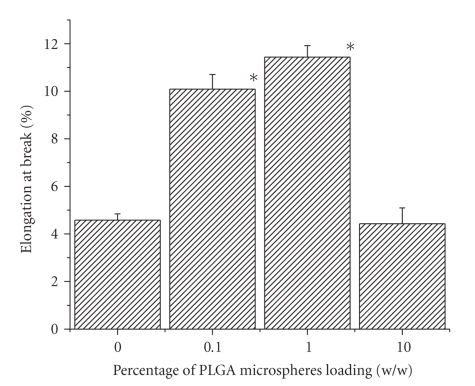
Effect of PLGA microsphere incorporation on percentage elongation at break of gelatin scaffold. Values are mean ± S.D. (*n* = 3). *P < .05, compared to gelatin scaffold having no PLGA microsphere.

PLGA microspheres loaded scaffolds were tested for tensile properties in dry condition. Tensile strength (see Figure 6) of 0.1% and 1% w/w PLGA microsphere loaded scaffolds (0.448 MPa and 0.406 MPa, resp.) was significantly higher than that of control (0.228 MPa) and among all, 0.1% has the highest tensile strength. Although tensile strength of 10% w/w PLGA microsphere loaded is higher (0.280 MPa) than that of control, the value was not significant. From the data, it was evident that there was no clear relation between the weight percentages of PLGA microspheres doping and tensile strength of the scaffold. Trend observed in percentage elongation at break is almost similar to the previous one, that is, percentage elongation at break for 0.1% and 1% w/w PLGA microsphere loaded scaffolds is significantly higher than that of control (see Figure 7). Percentage elongation at break for 0.1% and 1% w/w PLGA microspheres doped scaffold is 2.20 and 2.49 fold greater than that of control, where it is 0.96 fold less for 10% w/w PLGA doped scaffold.

### 3.7. Biodegradation

**Figure 8:**
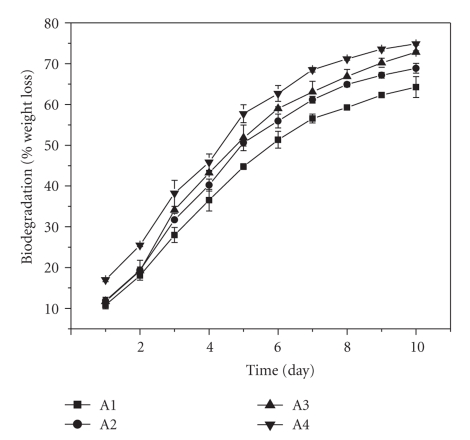
Effect of PLGA microsphere incorporation on biodegradation of gelatin scaffold. Degradation was studied for 10 days by incubating the scaffolds in PBS (pH-7.4) at 37°C. Values are mean ± S.D. (*n* = 3). (A1) Gelatin scaffold, (A2) 0.1% w/w PLGA microsphere loaded scaffold, (A3) 1% w/w PLGA microsphere loaded scaffold, and (A4) 10% w/w PLGA microsphere loaded scaffold ∗.

Biodegradation study showed that incorporation of microspheres in the scaffold up to a certain extent (up to 1% w/w) has no significant effect on early phase of degradation (see Figure 8). The extent of biodegradation after 48 hours was within 18-19% for all three scaffolds except 10% w/w PLGA microsphere loaded scaffold (25.6% biodegradation). However, with the progress of time, a variation in extent of biodegradation was observed. Result revealed that the rate and total extent of biodegradation were higher for scaffolds having higher PLGA microsphere content. This may be because of the time dependant degradation of PLGA itself by means of hydrolysis [23] which rendered the scaffold microenvironment acidic. Under these circumstances chemical intervention become predominant over the physical influence of the hydrophobic microsphere on scaffold degradation; therefore scaffolds having higher PLGA content showed higher degradation.

### 3.8. Study of Cell Adhesion

**Figure 9:**
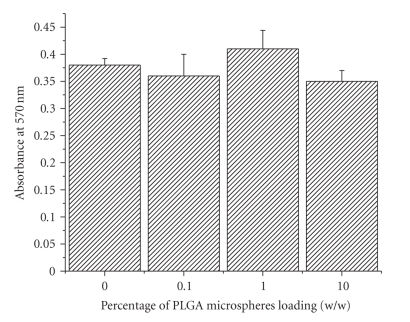
Effect of PLGA microsphere incorporation on cell adhesion after 4 hours of cell seeding. Values are mean ± S.D. (*n* = 3).

Cell adhesion study showed that incorporation has no significant effect on cell adhesion (see Figure 9). All the scaffolds showed comparable cell adhesion properties. This might be because the total number of microspheres present in the upper surface is not sufficient to exert any significant effect on cell adhesion.

### 3.9. Study of Cell Proliferation

**Figure 10:**
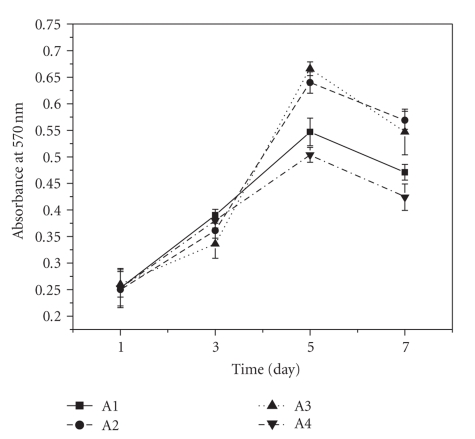
Effect of PLGA microsphere incorporation on murine L929 cells proliferation on gelatin scaffold. Proliferation was studied for 7 days. Values are mean ± S.D. (*n* = 3). (A1) Gelatin scaffold, (A2) 0.1% w/w PLGA microsphere loaded scaffold, (A3) 1% w/w PLGA microsphere loaded scaffold, and (A4) 10% w/w PLGA microsphere loaded scaffold. *P < .05, compared to gelatin scaffold having no PLGA microsphere.

Cell proliferation study (see Figure 10) showed that there was not much variation in cell proliferation for the first three days of culture. Growth of the cells on all three types scaffolds loaded with varying amount of PLGA microspheres was similar to that of control. Highest cell proliferation was achieved at day 5 for all type of scaffolds; however at day 5, the extent of cell proliferation on 0.1% and 1% w/w PLGA microspheres loaded scaffolds was significantly higher than that of control; on the other hand cell proliferation on 10% w/w PLGA microsphere incorporated scaffold was less than not only to that of 0.1% and 1% w/w PLGA microspheres loaded scaffold but even significantly less than the control. The same trend was observed up to day 7 but extent of proliferation was decreased compared to day 5 for all set of scaffolds.

### 3.10. Study of Cell Morphology

**Figure 11:**
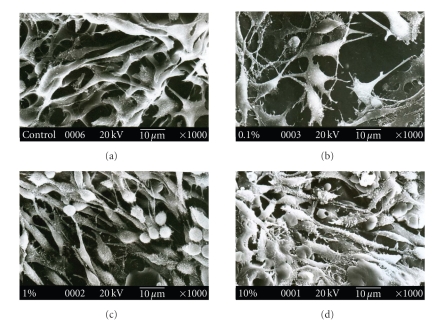
Scanning electron micrograph of mouse L929 cells cultured on PLGA microsphere incorporated gelatin scaffold after 3 days of initial seeding. (a) Gelatin scaffold, (b) 0.1% w/w PLGA microsphere loaded scaffold, (c) 1% w/w PLGA microsphere loaded scaffold, and (d) 10% w/w PLGA microsphere loaded scaffold. *P < .05, compared to gelatin scaffold having no PLGA microsphere.

From the scanning electron micrographs of cells cultured on microspheres loaded scaffold (see Figure 11), it was evident that microspheres have no adverse effect on cell growth. However, a stricken observation was that cells cultured on 10% w/w PLGA microsphere loaded scaffold has an elongated structure compared to the cells cultured on other scaffolds.

## 4. Discussion

Water molecules interacting with the hydrophobic surfaces do not form hydrogen bond with the surface. Instead, it forms a highly connected self-assembled structure using its own hydrogen bonding [24]. This exceptional behavior of water molecule on hydrophobic surfaces evoked the thought that presence of hydrophobic microspheres in natural hydrophilic polymer solution can alter the size and distribution of ice crystals formed at the time of freezing and thus can change the whole microarchitecture of the scaffold made by freeze drying. To verify this concept, we have taken PLGA microspheres incorporated gelatin scaffold as a model system. Prior to the fabrication of the model, the contact angle of PLGA and gelatin film was measured to confirm the difference in the hydrophobicity of two polymers used in the model. Result ensured that PLGA (65:35) is much hydrophobic than the gelatin used. During the fabrication of the model, care was taken to keep the size of the microspheres considerably lower (at least 5 times lower than the minimum pore size of the scaffold) than the pore sizes of the scaffold; otherwise, microspheres may block the pore structure. Microarchitecture analysis indicated that presence of PLGA microsphere during freeze drying actually reduced the pore size of scaffold. However in case of 1% w/w PLGA microsphere loaded scaffold, there were two sets of pores (150–120 *μ*m and 50–30 *μ*m). A probable explanation of such heterogeneity in pore size is the fusion of two or three adjacent pores by the rupture of common pore walls. This might be due to the steric effect of the microspheres embedded or because of strong water repelling force exerted by the hydrophobic microspheres. This assumption was strongly supported as 10% w/w PLGA microsphere loaded scaffold has no regular structure or pore size which varies from 30–150 *μ*m. Experimental result (see Figure 4) revealed that variations in swelling property of the scaffolds are not proportional with the amount of microspheres incorporation. Extent of swelling of 0.1% and 1% w/w PLGA microsphere loaded scaffolds was even higher than that of control (scaffold having no microsphere). However, 10% w/w PLGA microspheres incorporated scaffold has less swelling than control. This was contrary to our expectation that presence of greater amount of hydrophobic materials inside the scaffolds reduces the water up take to a greater extent. Probably incorporation of PLGA microspheres up to a certain extent in the scaffold enhanced its porosity that allows it to restore greater amount of water. Aforementioned assumption was supported by the trend observed in the variation in porosity (see Figure 5). Porosity data showed that porosity of 1% and 0.1% w/w microspheres loaded scaffolds was higher than that of control. This increased porosity might be because of higher interconnectivity of the 0.1% and 1% w/w PLGA microspheres incorporated scaffold as evident from the SEM micrographs (see Figure 3). Tensile strength of 0.1% and 1% w/w microsphere incorporated scaffold (see Figure 6) implied that presence of PLGA microspheres within the scaffold could enhance its mechanical strength. The same trend was observed in case of percentage elongation at break of those scaffolds. Higher values of percentage elongation at break in case of 0.1% and 1% w/w PLGA microsphere incorporated scaffold with respect to control and 10% w/w PLGA microsphere incorporated scaffold indicated the greater mechanical flexibility of 0.1% and 1% w/w PLGA microsphere incorporated scaffold. It was expected that presence of hydrophobic microspheres can contribute to the hydration of the scaffold thus can affect the initial phase of biodegradation but no significant difference in the rate of biodegradation was observed for first 48 hours in case of 0.1% and 1% w/w microspheres loaded scaffolds (see Figure 8). Although 10% PLGA microspheres incorporated scaffold had lower swelling properties, its high degradation rates indicate predominance of chemical factors over the extent of hydration. Cell adhesion study was aiming to characterize the influence of the presence of hydrophobic microspheres in the gelatin scaffold on cell adhesion. PLGA itself is a moderate substrate for cell adhesion [25], and it was expected that presence of hydrophobic PLGA microspheres inside the scaffold may play a critical role in early phase of cell adhesion because that phase of cell adhesion is primarily governed by simple electrostatic interaction between cells and substrate but result showed that all scaffolds have comparable cell adhesion property (see Figure 9). A probable explanation of this observation could be the presence of gelatin which is well known for its excellent cell adhesion property. Analysis of growth kinetics of L929 cells on PLGA microspheres incorporated scaffold revealed that microspheres have no direct influence on cell proliferation at the early phase of culture (up to day 3) but with time the effect become pronounced as evident from day 5 proliferation data. This may be because of the higher interconnectivity and porosity of 0.1 and 1% w/w PLGA microspheres loaded scaffold which leads to efficient diffusion of toxic products (produced by the cells) from the scaffold to the media rendering the scaffold microenvironment more hospitable to the cultured cells.

## 5. Conclusion

This effort has brought forward the consequence of PLGA microsphere incorporation during freeze drying of gelatin scaffold. Study has revealed that loading of hydrophobic PLGA microspheres in relatively hydrophilic gelatin scaffold not only change the scaffold microenvironment but also modulate other physical properties of the scaffold which influence the cell growth kinetics. The model gives an insight to the happening taken place inside the hydrophilic scaffold microenvironment due to the presence of microscale hydrophobic moieties at the time of scaffold fabrication by freeze-drying method. This study may find its application in designing a better-controlled release matrix for improved tissue engineering. Further investigation is required to find out the threshold difference in hydrophobicity of two polymers that may give rise to this kind of variation and effect of size variation of polymer spheres on scaffold's properties.

## Figures and Tables

**Table 1:** Effect of microsphere doping on scaffold microarchitecture. For each scaffold, 50 pores were analyzed to get the average pore size.

%age of PLGA microsphere doping (w/w) in gelatin	Average pore size (*μ*m)
0%	160
0.1%	110
1%	[150–120], [50–30]
10%	150–30
